# Crystal structure of triphenylphosphonium­meth­yl­enetrifluoroborate

**DOI:** 10.1107/S2056989017009884

**Published:** 2017-07-07

**Authors:** Christopher M. Bateman, Lev N. Zakharov, Eric R. Abbey

**Affiliations:** a Dept. of Chemistry, Biochemistry, and Physics, Eastern Washington University, Cheney, WA 99004, USA; b Department of Chemistry and Biochemistry, CAMCOR, University of Oregon, Eugene, OR 97403, USA

**Keywords:** crystal structure, tri­fluoro­borates, zwitterions, phospho­nium

## Abstract

The title compound, C_19_H_17_BF_3_P {alternative name: triphen­yl[(tri­fluoro­boran­yl)meth­yl]phosphanium}, was formed by the reaction of tri­phenyl­phosphine with potassium iodo­methyl­tri­fluoro­borate. The mol­ecule features a nearly staggered conformation along the P—C bond and a less than staggered conformation along the C—B bond. In the crystal, weak C—H⋯F hydrogen bonds between the *meta*-phenyl C—H groups and the tri­fluoro­borate B—F groups form chains of *R*_2_^2^(16) rings along [100]. These chains are are further stabilized by weak C—H⋯π inter­actions. A weak intra­molecular C—H⋯F hydrogen bond is also observed.

## Abstract 2

The synthesis, characterization and structural analysis of triphenylphosphoniummethylenetrifluoroborate are presented.

## Chemical context   

Alkyl­tri­phenyl­phospho­nium (Ph_3_P*RX*) salts are widely used as precursors in the preparation of phospho­rus ylides for Wittig-type olefination (Julia, 1985 ▸). Such olefination reactions continue to be one of the most important means of alkene generation. Potassium organotri­fluoro­borates (K*R*BF_3_) are common substrates used in Suzuki–Miyaura coupling as stable boronic acid precursors. Additionally, they may be used to produce organodihaloboranes (*R*B*X*_2_) (Darses & Genet, 2008 ▸). Seyferth & Grim (1961 ▸) showed that reaction of tri­phenyl­phosphine­methyl­ene ylide (Ph_3_PCH_2_^−^) with boron trifluoride di­ethyl­etherate (BF_3_-OEt_2_) yields triphen­yl[(tri­fluoro­boran­yl)meth­yl]phosphonium (Ph_3_PCH_2_BF_3_). We have synthesized Ph_3_PCH_2_BF_3_ *via* an alternate route, by reacting tri­phenyl­phosphine (PPh_3_) with potassium iodo­methyl­tri­fluoro­borate (ICH_2_BF_3_K) in 45% yield.
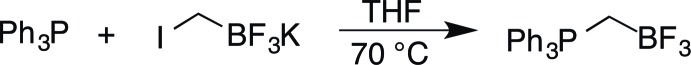


There are many examples of zwitterionic organotri­fluoro­borates containing ammonium moieties, but very few containing phospho­nium groups have been reported (see *Database survey*). Phospho­nium tri­fluoro­borates have been shown to enhance the hydrolytic stability of the *R*BF_3_ moiety (Wade *et al.*, 2010 ▸.) In this context we synthesized Ph_3_PCH_2_BF_3_ and report herein its crystal structure.
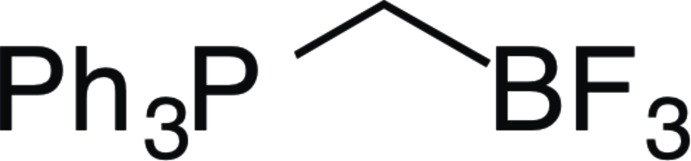


## Structural commentary   

**Figure 1:**
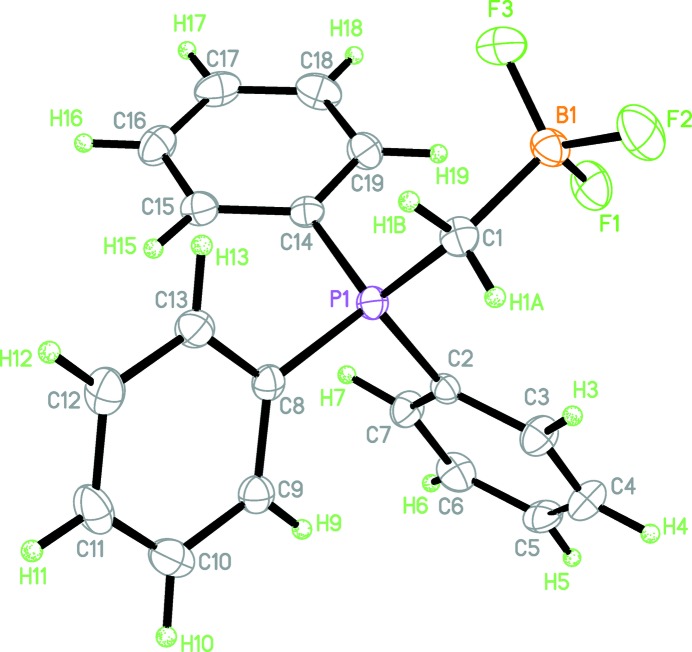
The mol­ecular structure of the title compound, with displacement ellipsoids drawn at the 50% probability level.

The mol­ecular structure of the title compound is shown in Fig. 1 ▸. A weak intra­molecular C—H⋯F hydrogen bond forms an *S*(7) ring (Table 1 ▸). The mol­ecule features a nearly *anti* conformation along the P1—C1 bond [B1—C1—P1—C8 torsion angle = 172.4 (2)°] and a less staggered conformation along the C1—B1 bond [F2—B1—C1—P1 torsion angle = 158.3 (2)°].

The B-F bond lengths fall within normal ranges for organotri­fluoro­borate compounds. The methyl­ene C—P bond length [1.787 (4) Å] and the C—B bond length [1.636 (4) Å] also fall within the normal range for similar compounds (Allen *et al.*, 1987 ▸). In terms of the surrounding angles, the B and P atoms appear to be *sp^3^* hybridized. The methyl­ene carbon is predominantly *sp^3^* hybridized, but has a distorted tetra­hedral geometry with a P1—C1—B1 angle of 119.7 (2)°.

## Supra­molecular features   

**Figure 2:**
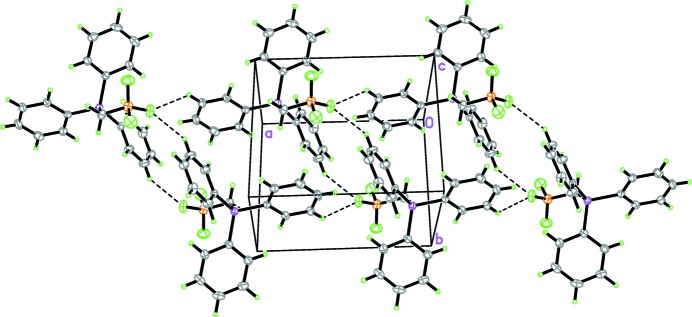
Part of the crystal structure, showing weak C—H⋯F hydrogen bonds as dashed lines.

**Figure 3:**
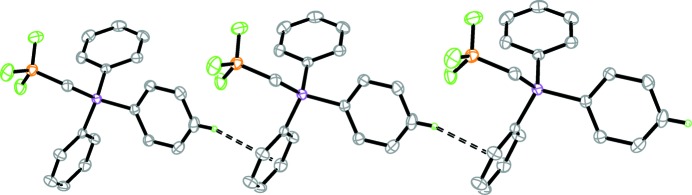
Part of the crystal structure, showing weak C—H⋯π inter­actions along [100] as dashed lines. Only the H atoms involved in these inter­actions are shown.

In the crystal, two weak C—H⋯F hydrogen bonds between the *meta* hydrogen atoms on the tri­phenyl­phospho­nium rings and the tri­fluoro­borate moiety (Table 1 ▸) fall within the range of distances observed in other tri­phenyl­phospho­nium tri­fluoro­borates (Wade *et al.*, 2010 ▸) and form chains of 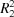
(16) rings along the [100] axis (Fig. 2 ▸). These chains are further stabilized by herringbone edge-to-face weak C—H⋯π inter­actions (Fig. 3 ▸).

## Database survey   

A search of the Cambridge Structural Database (Version 5.37, update February 2017; Groom *et al.*, 2016 ▸) for phospho­nium-containing tri­fluoro­borates yielded only five structures: FUYDIN (Wade *et al.*, 2010 ▸), OZOJOD (Gott *et al.*, 2011 ▸), PUXWEL (Piskunov *et al.*, 2010 ▸), ZEKLEI (Li *et al.*, 2012 ▸) and ZEKLOS (Zibo *et al.*, 2012 ▸).

## Synthesis and crystallization   

Potassium iodo­methyl­tri­fluoro­borate (1.00 g, 4.04 mmol) and tri­phenyl­phosphine (1.11 g, 4.23 mmol) were combined in a pressure flask containing a stir bar under nitro­gen, and anhydrous THF (25.0 mL) was added. The flask was sealed and heated to 343 K for 18 h. The reaction was cooled to room temperature and the solvent was removed *in vacuo.* The residue was washed with Et_2_O (3 x 10 mL) and the resulting solid was dissolved in a minimal amount of acetone and the product was precipitated with water and collected by filtration, to afford a white solid (0.63 g, 1.82 mmol, 45%.) X-ray quality crystals were grown by slow diffusion of pentane into a solution of the title compound dissolved in di­chloro­methane.

^1^H NMR (500 MHz, CDCl_3_) δ (ppm): 7.66 (*m*, 9H), 7.56 (*m*, 6H), 2.07 (*br d*, 2H, *J* = 15 Hz). ^13^C NMR (126 MHz, CDCl_3_) δ (ppm): 133.7 (*d*, *J* = 3 Hz), 133.5 (*d*, *J* = 10 Hz), 129.6 (*d*, *J* = 12 Hz) 123.2 (*d*, *J* = 87 Hz) (C—B *not* observed). ^11^B NMR (160 MHz, CDCl_3_) δ (ppm): 2.49 (*q*, *J* = 47 Hz). ^19^F NMR (470 MHz, CDCl_3_) δ (ppm): −138.9 (*q*, *J* = 37 Hz). FTIR (ATR, cm^−1^): 3070, 2960, 1587, 1484, 1438, 1146, 1104, 1025, 994, 969, 824, 754, 725, 691, 511, 497.

## Refinement details   

Crystal data, data collection and structure refinement details are summarized in Table 2 ▸. All H atoms were refined independently with isotropic displacement parameters.

## Supplementary Material

Crystal structure: contains datablock(s) I. DOI: 10.1107/S2056989017009884/lh5846sup1.cif

Structure factors: contains datablock(s) I. DOI: 10.1107/S2056989017009884/lh5846Isup2.hkl

Supporting information file. DOI: 10.1107/S2056989017009884/lh5846Isup3.cml

CCDC reference: 1560028

Additional supporting information:  crystallographic information; 3D view; checkCIF report

## Figures and Tables

**Table 1:** Hydrogen-bond geometry (Å, °) *Cg*1 is the centroid of the C2–C7 ring.

*D*—H⋯*A*	*D*—H	H⋯*A*	*D*⋯*A*	*D*—H⋯*A*
C4—H4⋯F1^i^	0.95 (3)	2.44 (2)	3.293 (4)	149.7 (17)
C12—H12⋯F1^ii^	0.96 (3)	2.37 (3)	3.084 (3)	131 (2)
C19—H19⋯F1	0.97 (2)	2.43 (2)	3.263 (3)	143.9 (18)
C11—H11⋯*Cg* ^ii^	0.89 (3)	2.77 (3)	3.639 (3)	165 (2)

Symmetry codes: (i) 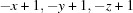; (ii) 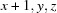.

**Table 2:** Experimental details

Crystal data	
Chemical formula	C_19_H_17_BF_3_P
*M*_r_	344.10
Crystal system, space group	Triclinic, *P* 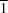
Temperature (K)	173
*a*, *b*, *c* (Å)	9.514 (2), 9.870 (3), 9.883 (3)
α, β, γ (°)	64.609 (6), 87.539 (7), 86.660 (7)
*V* (Å^3^)	836.8 (4)
*Z*	2
Radiation type	Mo *K*α
μ (mm^−1^)	0.19
Crystal size (mm)	0.13 × 0.07 × 0.01

Data collection	
Diffractometer	Bruker APEXII CCD
Absorption correction	Multi-scan (*SADABS*; Bruker, 2012 ▸)
*T*_min_, *T*_max_	0.925, 1.000
No. of measured, independent and observed [*I* > 2σ(*I*)] reflections	11811, 2953, 2090
*R*_int_	0.061
(sin θ/λ)_max_ (Å^−1^)	0.595

Refinement	
*R*[*F* ^2^ > 2σ(*F* ^2^)], *wR*(*F* ^2^), *S*	0.041, 0.094, 1.02
No. of reflections	2953
No. of parameters	285
H-atom treatment	All H-atom parameters refined
Δρ_max_, Δρ_min_ (e Å^−3^)	0.26, −0.29

Computer programs: *APEX2* and *SAINT* (Bruker, 2012 ▸), *SHELXS97* and *SHELXTL* (Sheldrick 2008 ▸) and *SHELXL2013* (Sheldrick 2015 ▸).

## supplementary crystallographic information

### Crystal data

**Table:**

C_19_H_17_BF_3_P	*Z* = 2
*M**_r_* = 344.10	*F*(000) = 356
Triclinic, *P*1	*D*_x_ = 1.366 Mg m^−^^3^
*a* = 9.514 (2) Å	Mo *K*α radiation, λ = 0.71073 Å
*b* = 9.870 (3) Å	Cell parameters from 2126 reflections
*c* = 9.883 (3) Å	θ = 2.3–23.9°
α = 64.609 (6)°	µ = 0.19 mm^−^^1^
β = 87.539 (7)°	*T* = 173 K
γ = 86.660 (7)°	Plate, colorless
*V* = 836.8 (4) Å^3^	0.13 × 0.07 × 0.01 mm

### Data collection

**Table:**

Bruker APEXII CCD diffractometer	2090 reflections with *I* > 2σ(*I*)
Radiation source: sealed tube	*R*_int_ = 0.061
φ and ω scans	θ_max_ = 25.0°, θ_min_ = 2.2°
Absorption correction: multi-scan (SADABS; Bruker, 2012)	*h* = −11→11
*T*_min_ = 0.925, *T*_max_ = 1.000	*k* = −10→11
11811 measured reflections	*l* = −11→11
2953 independent reflections	

### Refinement

**Table:**

Refinement on *F*^2^	0 restraints
Least-squares matrix: full	Hydrogen site location: difference Fourier map
*R*[*F*^2^ > 2σ(*F*^2^)] = 0.041	All H-atom parameters refined
*wR*(*F*^2^) = 0.094	*w* = 1/[σ^2^(*F*_o_^2^) + (0.0458*P*)^2^] where *P* = (*F*_o_^2^ + 2*F*_c_^2^)/3
*S* = 1.02	(Δ/σ)_max_ < 0.001
2953 reflections	Δρ_max_ = 0.26 e Å^−^^3^
285 parameters	Δρ_min_ = −0.29 e Å^−^^3^

### Special details

**Table:**

Geometry. All esds (except the esd in the dihedral angle between two l.s. planes) are estimated using the full covariance matrix. The cell esds are taken into account individually in the estimation of esds in distances, angles and torsion angles; correlations between esds in cell parameters are only used when they are defined by crystal symmetry. An approximate (isotropic) treatment of cell esds is used for estimating esds involving l.s. planes.

### Fractional atomic coordinates and isotropic or equivalent isotropic displacement parameters (Å^2^)

**Table:**

	*x*	*y*	*z*	*U*_iso_*/*U*_eq_	
P1	0.88100 (6)	0.18041 (7)	0.68950 (7)	0.01697 (18)	
B1	0.6884 (3)	0.2752 (3)	0.8793 (3)	0.0230 (7)	
F1	0.58103 (14)	0.26765 (16)	0.79007 (15)	0.0321 (4)	
F2	0.66153 (16)	0.40289 (16)	0.90564 (16)	0.0385 (4)	
F3	0.68558 (16)	0.14803 (16)	1.01567 (15)	0.0381 (4)	
C1	0.8421 (3)	0.2870 (3)	0.7950 (3)	0.0208 (6)	
C2	0.7769 (2)	0.2468 (2)	0.5241 (2)	0.0159 (5)	
C3	0.7246 (3)	0.3952 (3)	0.4576 (3)	0.0244 (6)	
C4	0.6496 (3)	0.4457 (3)	0.3269 (3)	0.0290 (6)	
C5	0.6268 (3)	0.3508 (3)	0.2611 (3)	0.0260 (6)	
C6	0.6785 (3)	0.2042 (3)	0.3260 (3)	0.0270 (6)	
C7	0.7526 (3)	0.1511 (3)	0.4578 (3)	0.0227 (6)	
C8	1.0645 (2)	0.1992 (2)	0.6332 (2)	0.0177 (5)	
C9	1.1081 (3)	0.2542 (3)	0.4834 (3)	0.0208 (6)	
C10	1.2509 (3)	0.2685 (3)	0.4467 (3)	0.0271 (6)	
C11	1.3487 (3)	0.2274 (3)	0.5571 (3)	0.0282 (6)	
C12	1.3064 (3)	0.1730 (3)	0.7066 (3)	0.0249 (6)	
C13	1.1648 (2)	0.1596 (3)	0.7452 (3)	0.0224 (6)	
C14	0.8521 (2)	−0.0170 (3)	0.7952 (2)	0.0175 (5)	
C15	0.9617 (3)	−0.1247 (3)	0.8201 (3)	0.0221 (6)	
C16	0.9361 (3)	−0.2760 (3)	0.8966 (3)	0.0276 (6)	
C17	0.8011 (3)	−0.3208 (3)	0.9478 (3)	0.0296 (6)	
C18	0.6919 (3)	−0.2144 (3)	0.9237 (3)	0.0293 (6)	
C19	0.7161 (3)	−0.0636 (3)	0.8475 (3)	0.0234 (6)	
H1A	0.860 (3)	0.389 (3)	0.727 (3)	0.034 (8)*	
H1B	0.913 (3)	0.256 (3)	0.864 (3)	0.036 (8)*	
H3	0.736 (3)	0.457 (3)	0.510 (3)	0.048 (8)*	
H4	0.616 (2)	0.547 (3)	0.282 (2)	0.024 (7)*	
H5	0.578 (2)	0.385 (3)	0.171 (3)	0.029 (7)*	
H6	0.662 (3)	0.139 (3)	0.281 (3)	0.032 (7)*	
H7	0.785 (2)	0.043 (3)	0.508 (2)	0.022 (6)*	
H9	1.043 (2)	0.282 (2)	0.404 (2)	0.019 (6)*	
H10	1.279 (2)	0.310 (3)	0.342 (3)	0.030 (7)*	
H11	1.441 (3)	0.233 (3)	0.535 (3)	0.028 (7)*	
H12	1.374 (3)	0.148 (3)	0.784 (3)	0.039 (8)*	
H13	1.135 (2)	0.119 (2)	0.851 (3)	0.024 (6)*	
H15	1.055 (2)	−0.099 (2)	0.789 (2)	0.020 (6)*	
H16	1.014 (3)	−0.352 (3)	0.915 (3)	0.036 (7)*	
H17	0.785 (3)	−0.429 (3)	1.000 (3)	0.042 (8)*	
H18	0.603 (3)	−0.246 (3)	0.958 (3)	0.032 (7)*	
H19	0.639 (2)	0.011 (3)	0.829 (2)	0.023 (6)*	

### Atomic displacement parameters (Å^2^)

**Table:**

	*U*^11^	*U*^22^	*U*^33^	*U*^12^	*U*^13^	*U*^23^
P1	0.0150 (3)	0.0181 (4)	0.0176 (3)	−0.0004 (2)	−0.0011 (2)	−0.0073 (3)
B1	0.0240 (16)	0.0278 (18)	0.0209 (15)	0.0002 (13)	−0.0003 (12)	−0.0141 (13)
F1	0.0175 (7)	0.0438 (10)	0.0395 (9)	0.0008 (6)	−0.0033 (6)	−0.0220 (8)
F2	0.0425 (9)	0.0369 (10)	0.0466 (10)	−0.0011 (7)	0.0079 (7)	−0.0287 (8)
F3	0.0475 (10)	0.0350 (9)	0.0237 (8)	0.0007 (7)	0.0056 (7)	−0.0058 (7)
C1	0.0199 (13)	0.0252 (16)	0.0203 (13)	−0.0003 (11)	−0.0050 (11)	−0.0123 (12)
C2	0.0132 (12)	0.0185 (14)	0.0163 (12)	−0.0006 (10)	0.0006 (9)	−0.0078 (10)
C3	0.0286 (14)	0.0200 (15)	0.0252 (13)	−0.0005 (11)	−0.0048 (11)	−0.0100 (12)
C4	0.0344 (16)	0.0198 (16)	0.0270 (14)	0.0022 (12)	−0.0058 (12)	−0.0044 (12)
C5	0.0255 (14)	0.0309 (17)	0.0167 (13)	−0.0010 (12)	−0.0047 (11)	−0.0051 (12)
C6	0.0273 (14)	0.0368 (17)	0.0250 (14)	−0.0013 (12)	−0.0035 (11)	−0.0205 (13)
C7	0.0283 (14)	0.0198 (15)	0.0220 (13)	0.0021 (11)	−0.0040 (11)	−0.0108 (12)
C8	0.0173 (12)	0.0140 (13)	0.0212 (12)	−0.0012 (10)	−0.0003 (10)	−0.0069 (10)
C9	0.0215 (13)	0.0191 (14)	0.0224 (13)	−0.0003 (10)	−0.0006 (11)	−0.0093 (11)
C10	0.0301 (15)	0.0254 (16)	0.0269 (15)	−0.0053 (12)	0.0085 (12)	−0.0124 (12)
C11	0.0174 (14)	0.0246 (16)	0.0433 (17)	−0.0053 (11)	0.0070 (13)	−0.0153 (13)
C12	0.0182 (14)	0.0222 (15)	0.0332 (15)	−0.0034 (11)	−0.0032 (12)	−0.0102 (12)
C13	0.0190 (13)	0.0228 (14)	0.0221 (13)	−0.0027 (10)	0.0001 (11)	−0.0063 (11)
C14	0.0188 (12)	0.0195 (14)	0.0152 (12)	−0.0023 (10)	−0.0021 (9)	−0.0081 (10)
C15	0.0210 (14)	0.0238 (15)	0.0205 (13)	−0.0042 (11)	−0.0007 (11)	−0.0082 (11)
C16	0.0343 (16)	0.0196 (15)	0.0277 (14)	0.0022 (12)	−0.0050 (12)	−0.0091 (12)
C17	0.0448 (18)	0.0187 (16)	0.0238 (14)	−0.0086 (13)	0.0007 (12)	−0.0070 (12)
C18	0.0292 (16)	0.0316 (17)	0.0293 (15)	−0.0142 (13)	0.0073 (12)	−0.0145 (13)
C19	0.0192 (13)	0.0257 (15)	0.0264 (14)	−0.0030 (12)	0.0012 (11)	−0.0120 (12)

### Geometric parameters (Å, º)

**Table:**

P1—C1	1.787 (2)	C8—C13	1.401 (3)
P1—C2	1.796 (2)	C9—C10	1.390 (3)
P1—C8	1.805 (2)	C9—H9	0.96 (2)
P1—C14	1.804 (2)	C10—C11	1.373 (4)
B1—F3	1.394 (3)	C10—H10	0.96 (2)
B1—F2	1.400 (3)	C11—C12	1.388 (4)
B1—F1	1.404 (3)	C11—H11	0.89 (2)
B1—C1	1.636 (4)	C12—C13	1.383 (3)
C1—H1A	0.96 (3)	C12—H12	0.95 (3)
C1—H1B	0.92 (3)	C13—H13	0.99 (2)
C2—C3	1.394 (3)	C14—C15	1.395 (3)
C2—C7	1.395 (3)	C14—C19	1.401 (3)
C3—C4	1.383 (3)	C15—C16	1.385 (3)
C3—H3	0.97 (3)	C15—H15	0.94 (2)
C4—C5	1.380 (4)	C16—C17	1.385 (4)
C4—H4	0.95 (2)	C16—H16	0.99 (3)
C5—C6	1.377 (4)	C17—C18	1.385 (4)
C5—H5	0.94 (2)	C17—H17	0.99 (3)
C6—C7	1.385 (3)	C18—C19	1.378 (3)
C6—H6	0.95 (2)	C18—H18	0.92 (3)
C7—H7	1.00 (2)	C19—H19	0.97 (2)
C8—C9	1.394 (3)		
			
C1—P1—C2	111.67 (12)	C9—C8—C13	119.8 (2)
C1—P1—C8	108.25 (11)	C9—C8—P1	122.17 (18)
C2—P1—C8	108.48 (10)	C13—C8—P1	118.04 (17)
C1—P1—C14	113.04 (12)	C10—C9—C8	119.6 (2)
C2—P1—C14	107.54 (10)	C10—C9—H9	117.9 (13)
C8—P1—C14	107.70 (11)	C8—C9—H9	122.5 (13)
F3—B1—F2	109.0 (2)	C11—C10—C9	120.4 (2)
F3—B1—F1	108.4 (2)	C11—C10—H10	121.1 (14)
F2—B1—F1	108.5 (2)	C9—C10—H10	118.5 (14)
F3—B1—C1	110.9 (2)	C10—C11—C12	120.6 (2)
F2—B1—C1	109.1 (2)	C10—C11—H11	121.3 (15)
F1—B1—C1	110.84 (19)	C12—C11—H11	118.1 (15)
B1—C1—P1	119.66 (17)	C13—C12—C11	119.9 (2)
B1—C1—H1A	111.2 (15)	C13—C12—H12	119.0 (15)
P1—C1—H1A	105.1 (15)	C11—C12—H12	121.1 (15)
B1—C1—H1B	110.3 (16)	C12—C13—C8	119.8 (2)
P1—C1—H1B	103.3 (16)	C12—C13—H13	120.1 (13)
H1A—C1—H1B	106 (2)	C8—C13—H13	120.1 (13)
C3—C2—C7	119.4 (2)	C15—C14—C19	119.2 (2)
C3—C2—P1	120.63 (17)	C15—C14—P1	121.19 (17)
C7—C2—P1	119.90 (17)	C19—C14—P1	119.50 (18)
C4—C3—C2	119.7 (2)	C16—C15—C14	120.3 (2)
C4—C3—H3	122.1 (16)	C16—C15—H15	117.2 (14)
C2—C3—H3	118.0 (16)	C14—C15—H15	122.5 (14)
C5—C4—C3	120.6 (3)	C17—C16—C15	120.0 (3)
C5—C4—H4	120.7 (14)	C17—C16—H16	120.0 (15)
C3—C4—H4	118.7 (14)	C15—C16—H16	120.0 (15)
C6—C5—C4	120.0 (2)	C16—C17—C18	120.1 (3)
C6—C5—H5	118.9 (15)	C16—C17—H17	118.8 (15)
C4—C5—H5	121.1 (15)	C18—C17—H17	121.2 (15)
C5—C6—C7	120.3 (2)	C19—C18—C17	120.4 (3)
C5—C6—H6	119.8 (15)	C19—C18—H18	120.3 (16)
C7—C6—H6	119.9 (15)	C17—C18—H18	119.2 (16)
C6—C7—C2	120.0 (2)	C18—C19—C14	120.0 (2)
C6—C7—H7	120.3 (13)	C18—C19—H19	120.6 (13)
C2—C7—H7	119.6 (13)	C14—C19—H19	119.4 (13)
			
F3—B1—C1—P1	81.6 (2)	C2—P1—C8—C13	177.28 (18)
F2—B1—C1—P1	−158.33 (17)	C14—P1—C8—C13	−66.6 (2)
F1—B1—C1—P1	−38.9 (3)	C13—C8—C9—C10	0.4 (3)
C2—P1—C1—B1	68.2 (2)	P1—C8—C9—C10	178.94 (18)
C8—P1—C1—B1	−172.42 (19)	C8—C9—C10—C11	0.6 (4)
C14—P1—C1—B1	−53.2 (2)	C9—C10—C11—C12	−0.8 (4)
C1—P1—C2—C3	25.8 (2)	C10—C11—C12—C13	0.1 (4)
C8—P1—C2—C3	−93.4 (2)	C11—C12—C13—C8	0.8 (4)
C14—P1—C2—C3	150.38 (18)	C9—C8—C13—C12	−1.1 (3)
C1—P1—C2—C7	−156.73 (19)	P1—C8—C13—C12	−179.71 (19)
C8—P1—C2—C7	84.1 (2)	C1—P1—C14—C15	−119.9 (2)
C14—P1—C2—C7	−32.2 (2)	C2—P1—C14—C15	116.40 (19)
C7—C2—C3—C4	−0.1 (4)	C8—P1—C14—C15	−0.3 (2)
P1—C2—C3—C4	177.32 (19)	C1—P1—C14—C19	63.0 (2)
C2—C3—C4—C5	−0.3 (4)	C2—P1—C14—C19	−60.7 (2)
C3—C4—C5—C6	0.2 (4)	C8—P1—C14—C19	−177.42 (18)
C4—C5—C6—C7	0.5 (4)	C19—C14—C15—C16	−0.4 (3)
C5—C6—C7—C2	−1.0 (4)	P1—C14—C15—C16	−177.49 (18)
C3—C2—C7—C6	0.8 (4)	C14—C15—C16—C17	0.4 (4)
P1—C2—C7—C6	−176.70 (18)	C15—C16—C17—C18	−0.5 (4)
C1—P1—C8—C9	−122.6 (2)	C16—C17—C18—C19	0.6 (4)
C2—P1—C8—C9	−1.3 (2)	C17—C18—C19—C14	−0.6 (4)
C14—P1—C8—C9	114.8 (2)	C15—C14—C19—C18	0.5 (3)
C1—P1—C8—C13	55.9 (2)		

### Hydrogen-bond geometry (Å, º)

Cg1 is the centroid of the C2–C7 ring.

**Table:**

*D*—H···*A*	*D*—H	H···*A*	*D*···*A*	*D*—H···*A*
C4—H4···F1^i^	0.95 (3)	2.44 (2)	3.293 (4)	149.7 (17)
C12—H12···F1^ii^	0.96 (3)	2.37 (3)	3.084 (3)	131 (2)
C19—H19···F1	0.97 (2)	2.43 (2)	3.263 (3)	143.9 (18)
C11—H11···*Cg*^ii^	0.89 (3)	2.77 (3)	3.639 (3)	165 (2)

Symmetry codes: (i) −*x*+1, −*y*+1, −*z*+1; (ii) *x*+1, *y*, *z*.
